# 

**Published:** 1892-01

**Authors:** 


					﻿Vol XII.
JANUARY, 1892.
No. 1.
THE
pOMtEOPATHlC pHY^lClAJfl,
A Monthly Journal of Homoeopathic Materia Medica
and Clinical Medicine.
“ He it a freeman whom truth makes free, and all are slaves beside."
"Seek the Truth: come whence it may, cost what it will."
EDITED AND PUBLISHED BY
WALTER M. JAMES, M. D.
PHILADELPHIA :
No. lies SPRUCK STREET).
X.O2Sr±>pKT :
ALFRED HEATH & CO., Sole Agents bob Gbeat Britain,
114 Ebury Street, Eaton Square.
•Yearly Subscription, $2.50. invariably in advance. Single numbers, 25 cts.
Entered st the Philadelphia Poet-Office aa Seoond-Clue Mail Batter.
				

